# Reliability of medical record abstraction by non-physicians for orthopedic research

**DOI:** 10.1186/1471-2474-14-181

**Published:** 2013-06-09

**Authors:** Michael Y Mi, Jamie E Collins, Vladislav Lerner, Elena Losina, Jeffrey N Katz

**Affiliations:** 1 Harvard Medical School, Harvard University, Boston, MA 02115, USA; 2 Orthopedic and Arthritis Center for Outcomes Research, Department of Orthopedic Surgery, Brigham and Women’s Hospital, Boston, MA 02115, USA; 3 Department of Biostatistics, Boston University School of Public Health, Boston, MA 02118, USA; 4 Division of Rheumatology, Immunology and Allergy, Brigham and Women’s Hospital, Boston, MA 02115, USA; 5 Medicine and Orthopedic Surgery, Harvard Medical School, Brigham and Women's Hospital, BC - 4th floor, 75 Francis Street, Boston, MA 02115, USA

**Keywords:** Medical record review, Reliability, Kappa statistic, Total knee replacement

## Abstract

**Background:**

Medical record review (MRR) is one of the most commonly used research methods in clinical studies because it provides rich clinical detail. However, because MRR involves subjective interpretation of information found in the medical record, it is critically important to understand the reproducibility of data obtained from MRR. Furthermore, because medical record review is both technically demanding and time intensive, it is important to establish whether trained research staff with no clinical training can abstract medical records reliably.

**Methods:**

We assessed the reliability of abstraction of medical record information in a sample of patients who underwent total knee replacement (TKR) at a referral center. An orthopedic surgeon instructed two research coordinators (RCs) in the abstraction of inpatient medical records and operative notes for patients undergoing primary TKR. The two RCs and the surgeon each independently reviewed 75 patients’ records and one RC reviewed the records twice. Agreement was assessed using the proportion of items on which reviewers agreed and the kappa statistic.

**Results:**

The kappa for agreement between the surgeon and each RC ranged from 0.59 to 1 for one RC and 0.49 to 1 for the other; the percent agreement ranged from 82% to 100% for one RC and 70% to 100% for the other. The repeated abstractions by the same RC showed high intra-rater agreement, with kappas ranging from 0.66 to 1 and percent agreement ranging from 97% to 100%. Inter-rater agreement between the two RCs was moderate with kappa ranging from 0.49 to 1 and percent agreement ranging from 76% to 100%.

**Conclusion:**

The MRR method used in this study showed excellent reliability for abstraction of information that had low technical complexity and moderate to good reliability for information that had greater complexity. Overall, these findings support the use of non-surgeons to abstract surgical data from operative notes.

## Background

Medical record review (MRR) is a commonly used method in clinical research to ascertain exposures (e.g. co-morbidities) or outcomes (e.g. complications) [1]. However, because medical records are meant to document care and are not designed as research tools, MRR poses several challenges in the research setting. Medical records may be incomplete, and the differential availability of information may result in misclassification and potential bias [1]. Medical information must be observed, recorded in the medical record, abstracted, coded, and analyzed; errors may occur at every step [2,3].

Problems with validity and reliability of MRR are generally recognized [4-7]. Inter-observer variability can vary widely in the abstraction of medical records by physician reviewers [8]. Currently, there is no official standard for reporting on the process used for MRR in clinical research as there is for meta-analysis research, such as the QUOROM statement [9]. Various proposed strategies for improvement such as abstraction monitoring and continuous abstractor training appear to be successful [10-12]. Nevertheless, it is impossible to ensure perfect validity and reliability; therefore, these parameters should be reported in MRR studies to provide context for interpreting the results. Physician review is expensive and consequently MRR studies are often carried out by researchers without medical training. The reliability of medical record abstraction by non-clinical personnel has received little study.

In this study, we evaluated the reproducibility of MRR in the context of studying risk factors for revision of total knee replacement (TKR). We assessed inter-rater reliability of MRR abstraction between a physician and two research coordinators (RCs) and between the two RCs; we also assessed the intra-rater (test-retest) reliability of a single RC.

## Methods

### Overview

This reliability study was conducted as part of a larger nested case-controlled study that examined risk factors for revision of TKR. The subjects of the study were 438 patients who received a primary TKR at a tertiary referral hospital between 1996 and 2009. Of these patients, 147 went on to have a revision TKR at the same institution or another sister tertiary referral hospital. The remaining patients (N = 291) did not have revision TKR and served as controls. Controls were matched to the cases based on surgery year and orthopedic surgeon. We developed an abstraction tool and used it to record medical record information on each subject. The tool included patient demographic information, medical history, social history, and prosthesis information. In particular, we abstracted details on the surgical procedures from the surgeons’ operative notes. The study was approved by the Partners HealthCare Human Subject Committee.

### Research coordinator training

Because operative notes contain medical jargon and technical language, an orthopedic surgeon taught the abstraction method to two research coordinators who had no formal clinical training. RC1 is a college graduate with no higher level degrees and two years of experience in clinical orthopedics research. RC2 attended college with no higher level degrees and one year of experience in clinical orthopedics research. First, the RCs and the surgeon reviewed charts together to learn the language and approach. Subsequently, during the pilot phase, the RCs independently reviewed charts, and their results were reviewed by the surgeon. Training was complete when the surgeon deemed the reviews to be accurate.

### Sub-study design

To test the reliability of this abstraction method and training process, we randomly selected 75 subjects from the larger study population. The sample size was chosen to ensure reasonable precision in the estimate of agreement statistics, such as the Cohen’s kappa. More specifically, given an *a priori* estimate of 75% agreement, a sample size of 75 provided a 95% C.I. around the point estimate of 65% to 85% agreement. To ensure that the abstractors were blinded to the data and had no prior exposure to the medical records, this study was carried out prior to the full data abstraction for the nested case–control parent TKR study.

Each patient’s operative note was reviewed four times: once by an orthopedic surgeon, once by one RC, and twice by another RC. This design permitted us to assess validity (agreement between the surgeon and each RC), inter-rater reliability (reproducibility between two RCs), and intra-rater reliability (reproducibility between the two abstractions by the same RC). Using the abstraction form developed for the parent TKR study, we created an abridged abstraction form to test validity and reliability (see Appendix). The form primarily addressed surgical techniques and bone deformities, as we were especially interested in the reliability of abstraction of the most technically sophisticated elements. Key words were appended to the form to guide the abstractor with the classification of data elements. The source of the information was Partners HealthCare’s Longitudinal Medical Record (LMR) system, which included radiological reports, pre-operative evaluation notes and operative notes.

### Statistical analysis

We combined response categories to create a new variable for some questions in order to improve clinical interpretation. Notably, “Lateral Release Performed” was combined with “Lateral Release Type” into a single new category, which had the options of “No”; “Yes – Patellar Tracking”; “Yes – Tibial Femoral Alignment”; “Yes – Both”; and “Insufficient Information”, which incorporated “Not Documented” (see Appendix). In addition, the “Bone Deformity” section was also simplified. Rather than splitting the categories of “Alignment” and “Predominant Compartment” by 3 different sources of information, a single category of “Alignment” and a single category of “Predominant Compartment” were created by combining information from the various sources, i.e., “D1a Alignment”, “D2a Alignment” and “D3a Alignment” combined to form one “Alignment” category (see Appendix).

The raters were de-identified for the analysis to minimize bias. We created two way tables for each pair of raters (six possible pairs) in each data category. To quantify intra- and inter-rater reliability, we calculated percent agreement and Cohen’s kappa coefficients with associated 95% confidence intervals based on the method described by Fleiss, *et al.*[13]. Cohen’s kappa is a statistical measure of agreement that is calculated based on expected vs. observed values and frequencies [14]. The formula for kappa is as follows:

$$\kappa =\left(p_{o}-p_{e}\right)/\left(1-p_{e}\right)$$

where *p*_*o*_ is the observed percent agreement and *p*_*e*_ is the expected percent agreement. The value of kappa falls between 0 and 1, with numbers closer to 0 indicating low agreement and values closer to 1 indicating high agreement. While there is no standardized guideline for the kappa value that constitutes acceptable agreement, Landis and Koch recommend the following categorization as shown in Table 1[15]:

**Table 1:** Categorization of different levels of Kappa by strength of agreement

**Kappa**	**Strength of agreement**
< 0	Poor
0 – 0.2	Slight
0.2 – 0.4	Fair
0.4 – 0.6	Moderate
0.6 – 0.8	Substantial
0.8 – 1	Almost perfect

Kappa is a useful statistical measure because it corrects for agreement that may arise based on chance alone; however, the kappa statistic can be biased by the distribution of agreement (see Discussion for further explanation). Therefore, we calculated kappa as well as percent agreement for the intra-rater agreement (same RC twice) and inter-rater agreement (between each RC and the expert clinician as well as between the two RCs). All statistical analyses were carried out using SAS v9.2 (SAS Inc., Carry, NC) and R (http://www.r-project.org).

## Results

To ensure that the random sample of patients for the reliability study was representative of the larger sample chosen for the parent TKR study, we compared the two samples. As shown in Table 2, the reliability sample (n = 75) was similar to subjects from the rest of the parent population (n = 363) in terms of age at primary TKR surgery, gender, race, marital status, and the operating orthopedic surgeon. A higher proportion of patients in the reliability study than in the control sample had a revision.

**Table 2:** Comparison of demographic information of patients selected for the reliability study versus that of all the patients from the risk factors for TKR revision study

**Categories**		**Sample patients**	**Other patients**
		**(n = 75)**	**(n = 363)**
Age	Mean	72.2	73.0
	SD	12.6	13.3
Age at primary TKR	Mean	62.4	62.9
	SD	12.9	12.7
Gender	Female	52 (69%)	255 (70%)
Race (%)	Asian	1 (1%)	1 (0%)
	Black	10 (13%)	46 (13%)
	Hispanic	3 (4%)	10 (3%)
	White	57 (76%)	293 (81%)
Marital Status (%)	Divorced	8 (11%)	31 (9%)
	Married	40 (53%)	192 (53%)
	Single	13 (17%)	54 (15%)
	Widowed	10 (13%)	72 (20%)
Revision? (%)	Yes	36 (48%)	111 (31%)
Surgeon (%)	Surgeon 1	18 (24%)	69 (19%)
	Surgeon 2	14 (19%)	50 (14%)
	Surgeon 3	9 (12%)	45 (12%)
	Surgeon 4	7 (9%)	47 (13%)
	Surgeon 5	7 (9%)	26 (7%)
	Other (15)	20 (27%)	126 (35%)

In Table 3, we show the final categories and each reviewer’s tabulations. Inter-rater agreement between the RCs and the surgeon was very good overall with kappa ranging from 0.49 to 1 and percent agreement from 70.4% to 100% (Table 4). In the cases of “Cement Fixation” for RC1 vs. RC2 and RC1 vs. RC1, the agreement was perfect, and “Yes” was selected for all patients; therefore, kappa was not calculable (Tables 3, 5 and 6). For RC1, there were moderate levels of agreement with the surgeon based on kappa of 0.59 for arthroplasty approach type and kappa of 0.66 for the predominant knee compartment (Table 4). The rest of the categories had substantial to perfect levels of agreement. RC2 had somewhat lower levels of kappa and percent agreement with the surgeon than RC1 (Table 4). The items for which RC2 had the highest levels of agreement with surgeon’s evaluation were the same as those for which RC1 had high agreement with the surgeon: index knee, bilateral operation, lateral release type, and whether the posterior cruciate ligament (PCL) was recessed. RC2 had moderate agreement with the surgeon in the more technical categories of arthroplasty approach type, alignment of knee, and predominant compartment of disease, with kappas of 0.49, 0.53, and 0.53, respectively.

**Table 3:** MRR categories and reviewers’ tabulations

**Categories/choices**	**Surgeon**	**RC1 (1st)**	**RC1 (2nd)**	**RC2**
**Index knee**				
Left	34	34	35	33
Right	40	40	39	40
**Bilateral operation**				
Yes	10	10	10	10
No	64	64	64	61
**Arthroplasty approach type**				
Medial/median peripatellar	70	71	71	73
Lateral peripatellar	0	0	0	0
Subvastus/midvastus	3	1	1	1
Quadriceplasty	0	0	0	0
Tibial Tubercle Osteotomy/TTO	0	0	0	0
Other	0	1	2	0
Not documented	0	1	0	0
**Cement fixation**				
Cemented	72	74	74	68
Cementless	1	0	0	0
Not documented				
**Lateral release type**				
No	56	56	56	56
Yes – patellar tracking	9	10	10	9
Yes – tibial femoral alignment	4	5	5	4
Yes – both patellar tracking and tibial femoral alignment	3	3	3	2
Not documented/insufficient information	1	0	0	2
**Posterior cruciate ligament recession**				
No	51	55	55	53
Yes	21	19	19	18
Not documented	1	0	0	2
**Knee alignment**				
Varus	49	43	43	34
Valgus	19	18	18	13
Neutral	3	7	7	3
Not documented/insufficient information	3	6	6	21
**Knee predominant compartment**				
Medial	49	46	46	44
Lateral	19	18	18	12
Even	3	2	2	4
Not documented/insufficient information	3	8	8	11

**Table 4:** Inter-rater agreement, surgeon vs. RC1 (1st abstraction) and RC2

	**Data elements**	**Kappa**	**95% CI**	**% Agreement**
RC1	Index knee	1.00	(1.00, 1.00)	100
	Bilateral operation	1.00	(1.00, 1.00)	100
	Arthroplasty approach type	0.59	(0.19, 0.99)	97.3
	Cement fixation	0	(0.00, 0.00)	98.6
	Lateral release type	0.93	(0.83, 1.00)	97.3
	Posterior cruciate ligament recession	0.86	(0.74, 0.99)	94.5
	Knee alignment	0.80	(0.68, 0.92)	89.2
	Knee predominant compartment	0.66	(0.51, 0.81)	82.4
RC2	Index knee	1.00	(1.00, 1.00)	100
	Bilateral operation	1.00	(1.00, 1.00)	100
	Arthroplasty approach type	0.49	(0.00, 1.00)	97.3
	Cement fixation	0	(0.00, 0.00)	98.5
	Lateral release type	0.68	(0.50, 0.85)	86.3
	Posterior cruciate ligament recession	0.74	(0.57, 0.90)	88.9
	Knee alignment	0.53	(0.39, 0.66)	70.4
	Knee predominant compartment	0.53	(0.37, 0.69)	74.6

**Table 5:** Inter-rater agreement, RC1 (1st abstraction) vs. RC2

**Data elements**	**Kappa**	**95% CI**	**% Agreement**
Index knee	1.00	(1.00, 1.00)	100
Bilateral operation	1.00	(1.00, 1.00)	100
Arthroplasty approach type	0.49	(0.00, 1.00)	97.3
Cement fixation	-	-	100
Lateral release type	0.66	(0.49, 0.83)	86.3
Posterior cruciate ligament recession	0.79	(0.64, 0.95)	91.8
Knee alignment	0.66	(0.52, 0.79)	77.5
Knee predominant compartment	0.65	(0.49, 0.80)	80.3

**Table 6:** Intra-rater agreement, RC1 (1st abstraction) vs. RC1 (2nd abstraction)

**Data elements**	**Kappa**	**95% CI**	**% Agreement**
Index knee	0.97	(0.92, 1.00)	98.6
Bilateral operation	1.00	(1.00, 1.00)	100
Arthroplasty approach type	0.66	(0.21, 1.00)	97.3
Cement fixation	-	-	100
Lateral release type	1.00	(1.00, 1.00)	100
Posterior cruciate ligament recession	1.00	(1.00, 1.00)	100
Knee alignment	1.00	(1.00, 1.00)	100
Knee predominant compartment	1.00	(1.00, 1.00)	100

We found that the inter-observer reliability between RC1 and RC2 was better than that between each of the RCs and the surgeon (Tables 4 and 5). The intra-rater agreement for RC1 was very good as demonstrated by kappas ranging from 0.66 to 1 and percent agreement from 97.3% to 100%. With the exception of index knee and arthroplasty approach type, there was perfect agreement between RC1’s first and second abstraction for all other variables (Table 6). Index knee had almost perfect agreement (98.6%). Arthroplasty approach type also had a high percent agreement (97.3%), and a substantial kappa of 0.66.

## Discussion

We examined the validity and the intra- and inter-rater reliabilities of abstraction of operative notes in a study of patients who underwent TKR. The findings suggest that trained research staff without prior clinical knowledge and experience can abstract medical records reliably and accurately. We found that both inter- and intra-rater reliability analyses showed almost perfect percent agreement and kappa values ranged from moderate to almost perfect depending on the type of data category. Simple data elements—the knee on which the TKR was performed and whether both knees were operated on at the same time—had almost perfect agreement. On the other hand, complex categories that require interpretation of how the surgery was conducted, such as the type of arthroplasty approach or the knee deformity, had lower agreement. Our results were consistent with previous findings, which have shown that demographic data (e.g. gender, age, etc.) typically have higher kappa than narrative text data looking for a key word (e.g. presence of a symptom) and that data requiring judgment have the lowest kappa [10,16,17]. Even for the most technical items, however, agreement between the RCs and the surgeons and between the two RCs was moderate to substantial.

One noteworthy aspect of the results is that certain categories had kappa values that seemed disproportionately low given the high percent agreement. This can be explained by the paradox of low Cohen’s kappa in the setting of high percent agreement—as can be seen for cement fixation and arthroplasty approach type. As seen in Table 3, nearly every patient was rated as having received a “Medial/Median Peripatellar” arthroplasty approach type. Consequently, the expected agreement is very high, and the formula for calculating kappa creates a large decrease in kappa for a relatively smaller decrease in percent agreement. As Kraemer wrote when she first reported this problem, a measurement method may have poor kappas simply because of the lack of variability in the population and not because of the intrinsic inaccuracy of the measurement method itself [18]. In essence, if the prevalence of a trait is very rare (or exceedingly common), then the expected agreement becomes so large that it is difficult to document reliability. Feinstein and Cicchetti further explored this issue and proposed that the kappa should be accompanied by additional information, such as percent agreement, to describe the degree to which a given kappa is biased [19,20]. In this paper, we followed their recommendations and reported both kappa and percent agreement.

In an analysis of the American College of Surgeons National Surgical Quality Improvement Program, Shiloach *et al*. reported comprehensively the inter-rater agreement for numerous chart abstraction categories, which provides a good basis of comparison for the inter-rater agreement documented in this study [12]. Shiloach *et al.* reported kappas for a range of dichotomous variables, which ranged from fair (0.32) to almost perfect (0.93). Variables with the lowest kappas were: do not resuscitate (DNR) status (0.32), history of angina (0.32), rest pain (0.38) and bleeding disorder (0.38). The percent agreement for these variables ranged from 94-99%, showing that, as in our study, low kappas may arise from high levels of chance agreement in studies of the reliability of medical record review [12].

This study had a few limitations in its design. First, we treated the surgeon’s MRR abstraction data as the “gold standard.” However, the abstractions of clinicians are not perfectly reliable [8]. Clinicians may introduce clinical judgment into the abstraction, potentially distorting results. On the other hand, research assistants are taught a standardized abstraction that is entirely objective and may be more reliable on that basis. Repeating this project with multiple surgeons and multiple research assistants would help clarify this issue. In addition, to more robustly measure reliability for all aspects of surgical information, more variables should be compared. Last, it is important to note that this study assumed that the information in the medical records was accurate and complete, which we could not assess.

The conclusions of any scientific study rely heavily on the assumption that the data collection process is both valid and reliable. In an effort to assess the quality of MRR studies, Gilbert *et al.* examined use of methodological features that may maximize validity and reliability. They identified eight possible strategies: proper training of abstractors, explicit case selection protocols, precisely defined variables, standardized abstraction forms, periodic review meetings to resolve problems, monitoring of abstractor performance, blinding chart reviewers to the hypothesis and group assignment, and testing inter-rater agreement [2]. Among 986 published studies reviewed, only 5% mentioned testing inter-rater reliability, and 0.4% reported the results of testing inter-rater reliability. Ten years later in a follow-up study, Worster *et al*. reported that inter-rater reliability was mentioned 22% of the time and tested 13% of the time [21]. Although these studies show some improvement in frequency of reported inter-rater reliability analysis, this remains an underreported (and perhaps underperformed) aspect of MRR research [22,23]. We hope that our study will contribute to the increased reporting of the quality of data collected for clinical research. To the best of our knowledge, this is the first assessment of agreement between clinically trained and clinically-untrained medical record reviewers. We cannot be certain, however, whether the paucity of studies of this issue simply reflects failure of authors of reliability studies to report the clinical training of the reviewers, or whether the question has not been addressed. To date, research has mainly addressed the interrater reliability of clinicians vs. non-clinicians and researchers of various levels of clinical experience when evaluating patients prospectively [24,25].

## Conclusions

Obtaining research data via medical record review involves multiple steps, each of which can introduce errors. Therefore, research that involves MRR should provide reasonable assurance that the data are valid and reliable. In this study, we assessed the reliability of a MRR method to abstract surgical information from TKR procedures. We found that the MRRs performed by research coordinators were reliable (inter- and intra-rater reliability) and valid (agreement with an orthopedic surgeon). Furthermore, our result was similar to that obtained from a nation-wide MRR survey of patients undergoing surgery [12]. The findings of this study provide support for the reliability and validity of MRR in the setting of research on risk factors for revision of TKR.

## Appendix

### Reliability Study Primary TKR Chart Abstraction Tool

A. Administrative

A1. Chart Review Date: __________ A2. Chart Reviewer: __________

B. Patient Information

B1. MRN (last 4 digits): _______________________

B2. Index Knee: ☐1. Left ☐ 2. Right

B2b. Bilateral: ☐1. Yes ☐ 2. No

C. Surgery

C1. Arthroplasty Approach Type:

☐ 1. Medial/Median Peripatellar (> = 90% Primary)

☐ 2. Lateral Peripatellar (<1% Primary, even less Revision)

☐ 3. Subvastus/Midvastus (<5% Primary, 0% Revision)

☐ 4. Quadriceplasty (<1% Primary; <20% Revision)

☐ 5. Tibial Tubercle Osteotomy/TTO (<1% Primary; <5% Revision, if quadriceplasty fails)

☐ 6. Other (Lateral Peripatellar, Quadriceplasty, Tibial Tubercle Osteotomy/TTO)

☐ 9. Not Documented (if approach not stated, then Medial/Median is implied)

C2. Fixation

☐ 1. Cemented (cement sticker exists or mentioned in LMR/Big Board/OpNotes)

☐ 2. Cementless

☐ 9. Not Documented

C3a. Lateral Release Performed

☐ 0. No (if good/smooth patella traction, or good varus/valgus stability after trial components, extremely unusual in varus knee)

☐ 1. Yes (i.e. Release of: lateral retinaculum/capsule, iliotibial band; popliteus; lateral/collateral ligament (LCL); pie crust technique)

☐ 9. Not Documented

C3b. Lateral Release Type

☐ 1. Patellar Tracking (C3a = Yes: i.e. Release of: lateral retinaculum/capsule)

☐ 2. Tibial Femoral Alignment (C3a = Yes: Valgus, iliotibial band; popliteus; lateral/collateral ligament (LCL); pie crust technique)

☐ 3. Both (C3a = Yes)

☐ 7. N/A (C3a = No/Not Documented)

☐ 8. Insufficient Information

C4. Post-cruciate (PCL) Recession Performed (if performed, likely to be mentioned)

☐ 0. No (if stated that knee is balanced/stable in flexion, flexion & extension gaps are equal, no lift-off evidence, recessed back to the proposed tibial articular osteotomy)

☐ 1. Yes (tight flexion gap; positive lift-off test)

☐ 8. N/A (if Constraint is not CR)

☐ 9. Not Documented

D. Bone Deformity

D1. Pre-Operative Surgeon Visit

D1a. Alignment D1b. Predominant Compartment

☐ 1. Varus ☐ 1. Medial

☐ 2. Valgus ☐ 2. Lateral

☐ 3. Neutral ☐ 3. Even

☐ 8. Insufficient Information ☐ 8. Insufficient Information

☐ 9. Not Documented ☐ 9. Not Documented

D2. LMR Operative Note

D2a. Alignment D2b. Predominant Compartment

☐ 1. Varus (osteophytes on medial side) ☐ 1. Medial

☐ 2. Valgus (anticipated if Lateral Release ☐ 2. Lateral

Performed = Yes, lateral wear in general,

i.e. deficiency in lateral femoral condyle;

drilling holes in lateral tibial plateau)

☐ 3. Neutral ☐ 3. Even

☐ 8. Insufficient Information ☐ 8. Insufficient Information

☐ 9. Not Documented ☐ 9. Not Documented

D3. X-Ray

D3a. Alignment D3b. Predominant Compartment

☐ 1. Varus ☐ 1. Medial

☐ 2. Valgus ☐ 2. Lateral

☐ 3. Neutral ☐ 3. Even

☐ 8. Insufficient Information ☐ 8. Insufficient Information

☐ 9. Not Documented ☐ 9. Not Documented

## Competing interests

The authors declare that they have no competing interests.

## Authors’ contributions

MM drafted the manuscript, collected data, and performed the statistical analysis. JC helped with drafting the manuscript and statistical analysis. SL collected data for the project and participated in the design. EL participated in the conception of the project and its design. JK conceived the project and its design and helped draft the manuscript. All authors read and approved the manuscript.

## Pre-publication history

The pre-publication history for this paper can be accessed here:

http://www.biomedcentral.com/1471-2474/14/181/prepub
